# Timing and intensity of proton pump inhibitor exposure hampers overall survival in patients with metastatic non-small cell lung cancer treated with immune checkpoint inhibitors: a retrospective cohort study

**DOI:** 10.3389/fimmu.2026.1682723

**Published:** 2026-01-28

**Authors:** Nuša Japelj, Nejc Horvat, Janja Jazbar, Mitja Kos, Veronika Pelicon Kapušin, Lea Knez

**Affiliations:** 1 Department of Social Pharmacy, Faculty of Pharmacy, University of Ljubljana, Ljubljana, Slovenia; 2 Department of Pharmacy, General Hospital »Dr. Franca Derganca« Nova Gorica, Šempeter pri Gorici, Slovenia; 3 Department of Pharmacy, University Clinic Golnik, Golnik, Slovenia

**Keywords:** carcinoma, non-small-cell lung, immune checkpoint inhibitors, proton pump inhibitors, survival analysis, gastrointestinal microbiome

## Abstract

**Introduction:**

Proton pump inhibitor (PPI) use has been associated with reduced immune checkpoint inhibitor (ICI) efficacy in metastatic non-small cell lung cancer (mNSCLC) with evidence limited to their use during a short time period around ICI initiation. This study evaluated the associations between the timing and intensity of PPI exposure up to one year before ICI initiation and overall survival (OS) in mNSCLC patients treated with ICIs.

**Methods:**

This retrospective cohort study included consecutive mNSCLC patients treated with ICIs within routine clinical practice. Patients were grouped by the timing of PPI exposure from 365 days before to 30 days after (−365 to +30 days) ICI initiation: (1) no PPIs within −365 to +30 days; (2) PPIs only within −365 to −31 days; and (3) PPIs also within ±30 days of ICI initiation. The intensity of PPI exposure was quantified with the total defined daily doses (DDDs). OS was estimated using Kaplan–Meier methods, and associations between PPI exposure and OS were analyzed using Cox proportional hazards models.

**Results:**

Of 391 patients included (median age 64.7 years, 58.6% male), 73.4% had access to PPI within −365 to +30 days of ICI initiation. PPI exposure within ±30 days (220 patients) was associated with reduced median OS (mOS) compared with no PPI exposure between −365 and +30 days of ICI initiation (mOS 15.4 vs 21.9 months; adjusted hazard ratio [aHR] 1.373, 95% CI 1.007–1.873, p = 0.045). High-intensity PPI exposure within −365 to +30 days of ICI initiation (DDD > 159; 108 patients) was also associated with reduced mOS compared with no PPI exposure in this period (mOS 13.4 vs 21.9 months; aHR 1.454, 95% CI 1.023–2.067, p = 0.037).

**Discussion:**

PPI use around ICI initiation as well as PPI treatment intensity over a wider period was associated with reduced OS. Efforts should be made to streamline PPI use.

## 1 Introduction

Lung cancer caused over 1.8 million deaths globally in 2022, accounting for almost a fifth of all cancer-related deaths in that year (1). Non-small cell lung cancer (NSCLC), which represents approximately 85% of lung cancer cases, is the predominant subtype driving this burden (2). Nearly half of the patients diagnosed with NSCLC present with metastatic disease (mNSCLC), and most of these cases lack oncogenic drivers that can be therapeutically targeted (3, 4).

Treatment options for non-oncogene-addicted mNSCLC were once limited to platinum-based chemotherapy, which achieved a median overall survival (mOS) of up to 14 months (5). However, the introduction of immune checkpoint inhibitors (ICIs) has transformed treatment paradigms in this patient population (6). Initially approved in 2015 for second-line use (7), ICIs rapidly transitioned to first-line treatment, either as monotherapy (8–10) or in combination with chemotherapy (11–14), becoming the standard of care for non-oncogene-addicted mNSCLC. In patients with programmed death ligand 1 (PD-L1) expression ≥ 50%, ICI monotherapy provides the greatest survival advantage, with near-doubling of mOS compared with chemotherapy alone (8, 9). More recently, ICIs have also demonstrated efficacy in the neoadjuvant (15), perioperative (16–18), and adjuvant (19) settings for resectable NSCLC, as well as in maintenance therapy for locally advanced disease (20). The benefit of ICIs has been well demonstrated in randomized clinical trials. Real-world data further support this benefit by showing encouraging improvements in mOS across broader, unselected mNSCLC populations after the integration of ICIs into routine clinical practice (21).

Despite these advances, ICIs fail to generate a response in almost two-thirds of mNSCLC patients (8–10). Resistance to ICIs is multifactorial and may involve tumor-intrinsic (e.g., PD-L1 expression, oncogenic mutations), tumor-microenvironment (e.g., immunosuppressive cells), and host-related characteristics (e.g., age, sex, gut microbiota composition) (22). Some of these factors are non-modifiable (e.g., age, sex, tumor biology), whereas others are potentially modifiable (e.g., gut microbiota composition, concomitant medicines) (23). Notably, the composition of gut microbiota has emerged as a particularly relevant and potentially modifiable biomarker influencing resistance to ICIs (22, 24, 25). In fact, gut microbiota supports antitumor immunity by promoting dendritic-cell activation and T-cell–mediated immune surveillance (24). Preclinical studies demonstrate that gut dysbiosis, whether induced by germ-free conditions or antibiotic exposure, impairs ICI efficacy in mice, whereas restoration of specific commensal species can reinstate therapeutic responses (24). Commonly prescribed medicines, particularly antibiotics and proton pump inhibitors (PPIs), can directly disrupt microbial diversity and favor the overgrowth of potentially pathogenic species (26, 27). Clinically, exposure to these medicines has been independently linked to reduced mOS in mNSCLC patients receiving ICIs (28–32). Antibiotic exposure near the time of ICI initiation as well as within the prior year has been associated with worse mOS in mNSCLC patients, with fluoroquinolones showing a dose-dependent relationship (28). For PPIs, strong evidence from *post hoc* analyses of randomized clinical trials indicates that PPI use within 30 days before or after initiating ICIs is associated with mOS being approximately halved (hazard ratio ≥ 1.45 compared with no PPI use) in mNSCLC patients (29–31). However, unlike antibiotics, the relevance of prolonged PPI exposure beyond this 30-day window, including the intensity of use, remains unclear.

Given the widespread use of PPIs among cancer patients (33) and increasing evidence that PPI exposure alters ICI efficacy, further investigation is warranted. These concerns and the above evidenced knowledge gaps provide the rationale for the present retrospective cohort study, in which the associations between the timing and intensity of PPI exposure up to one year before ICI initiation and OS in mNSCLC patients treated with ICIs were evaluated. It was hypothesized that PPI exposure within 30 days before or after ICI initiation, PPI exposure during the preceding year, and cumulative PPI intensity from the preceding year to 30 days after ICI initiation would each be associated with reduced OS.

## 2 Materials and methods

### 2.1 Study design

The study protocol for this retrospective cohort study was approved by the Medical Ethics Committee of the Republic of Slovenia (Protocol Number 0120-513/2021/6). Due to the retrospective design, the requirement for written informed consent was waived.

### 2.2 Setting and participants

The study was conducted at a single teaching hospital in Slovenia. It included consecutive adult patients (≥18 years at ICI initiation) with pathologically confirmed mNSCLC who received either immunotherapy as monotherapy (mono-IT) or a combination of chemotherapy and immunotherapy (chemo-IT), in any line of treatment. Patients were treated as part of routine clinical practice between July 2015 and December 2022.

Molecular testing was conducted routinely, adhering to standard laboratory guidelines and quality control procedures valid at the time of testing. PD-L1 testing was performed on formalin-fixed, paraffin-embedded histology samples or cytospins using PD-L1 monoclonal antibodies (22C3 clone, DAKO, Glostrup, Denmark). All patients were tested for epidermal growth factor receptor (EGFR) and anaplastic lymphoma kinase (ALK) alterations; after 2016, they were also tested for ROS proto-oncogene 1 (ROS1) molecular alterations. Kristen rat sarcoma oncogene (KRAS) status and PD-L1 expression were assessed in all patients. For first-line treatment, patients received mono-IT only if they had a PD-L1 expression ≥ 50%, according to the registrational status of the medicines, or a combination of ICI and platinum-based chemotherapy, irrespective of PD-L1 expression. In the second-line setting, patients were primarily treated with mono-IT. Chemo-IT was rarely used and mainly only in those with molecular alterations such as EGFR-activating mutations. Treatment decisions were guided by the oncologist’s discretion, following international clinical practice guidelines (34) and considering medicine availability constraints. Patients were followed up at a single institution, according to valid guidelines (34). Patient functional status was assessed using the Eastern Cooperative Oncology Group performance status (ECOG PS) scale (35).

Data on cancer treatment and survival outcomes were obtained from structured medical records. At this oncology center, all medical data relevant to oncology care are collected prospectively and in a standardized manner, using specifically designed pro-forms. Survival data were updated on October 21, 2024. Data on PPI exposure were obtained from electronic medication dispensing records. Patients were excluded if no records of dispensed medicines were available.

### 2.3 Data sources and variables

The primary outcome was OS, defined as the time from ICI initiation to death from any cause. The main exposures of interest were timing and intensity of PPI exposure in relation to ICI initiation.

#### 2.3.1 Timing of PPI exposure

The PPI exposure was determined based on dispensed prescriptions. The number of boxes and tablets was used to estimate whether patients had access to PPIs at a certain time point. Patients were considered exposed if they had access to at least one PPI tablet during the defined observation window. Patients were stratified by timing of PPI exposure into three groups: (1) no PPIs at −365 to +30 days of ICI initiation (patients who were not exposed to PPIs from 365 days before to 30 days after ICI initiation); (2) PPIs at −365 to −31 days of ICI initiation (patients who were exposed to PPIs exclusively in the pre-treatment period between 365 and 31 days before ICI initiation, without any exposure during the ±30-day window); and (3) PPIs at ±30 days of ICI initiation (patients who were exposed to PPIs during the 30 days before or after ICI initiation). These patients could also have had earlier PPI exposure.

#### 2.3.2 Intensity of PPI exposure

The intensity of PPI exposure was evaluated based on the total defined daily doses (DDDs) prescribed within the entire observation window (−365 to +30 days with ICI initiation as index day 0), as well as within two separate windows, namely, −365 to −31 days and ±30 days of ICI initiation. One DDD reflects the standardized daily average maintenance dose of a medicine for its primary indication in adults and was equivalent to a single dose of pantoprazole 40 mg, esomeprazole 30 mg, lansoprazole 30 mg, or omeprazole 20 mg (36).

For each patient, total DDDs were calculated within each observation window, and the median total DDDs are reported. Based on the total DDDs per patient, patients were grouped into three categories: (1) no PPI exposure (total DDD = 0); (2) low-intensity exposure (DDDs equivalent to an intake of up to 0.5 DDD per day [e.g., pantoprazole 20 mg, a lower maintenance dose], assuming 80% adherence); and (3) high-intensity exposure (DDDs equivalent to an intake of more than 0.5 DDD per day, assuming 80% adherence). For example, for the observation period from −365 to +30 days of ICI initiation, PPI DDD categories were calculated as follows: 0.5 × 396 × 0.80 = 159 DDDs, where 0.5 represents 0.5 DDD per day, 0.80 accounts for 80% adherence, and 396 corresponds to the total days in the defined period. These calculations resulted in the following DDD categories: DDD = 0 (no PPI exposure), DDD = 0.1–159 (low-intensity exposure), and DDD > 159 (high-intensity exposure). The 80% adherence threshold was applied to reflect typical adherence levels in chronic medicine use, which is commonly used in pharmacoepidemiologic research as a benchmark for adequate long-term therapy adherence (37). In addition to categorical grouping, total DDDs were also analyzed as a continuous variable.

### 2.4 Study size

A sample size of 152 patients per group (with vs without PPI exposure within ±30 days of ICI initiation) was required to detect a statistically significant hazard ratio (HR) of 1.45 for mOS with 80% power, assuming a control group mOS of 15 months, a 60-month accrual period, and a minimum follow-up of 12 months (29).

### 2.5 Statistical methods

Descriptive statistics were used to summarize baseline patient and treatment characteristics. Kaplan-Meier curves estimated OS, with 95% confidence intervals (CIs). Median follow-up time was calculated using the reverse Kaplan–Meier method, which treats censoring times as events and actual events (e.g., death) as censored. Associations between PPI exposure and mOS were assessed using Cox proportional hazards models in univariable and multivariable analyses. Multivariable models were adjusted for key prognostic factors, including sex, age, body mass index, ECOG PS, cancer histology (non-squamous, squamous), the presence of brain or liver metastases, PD-L1 expression (%), therapy line (first, second or more), and systemic cancer therapy type (mono-IT, chemo-IT). Analyses were conducted for the total cohort. All statistical analyses were performed using SPSS version 29.0, while figures were generated in R version R 4.3.2. For patients with missing data on cancer treatment, no data imputation was performed. This study was reported in accordance with the Strengthening the Reporting of Observational Studies in Epidemiology (STROBE) guidelines (38).

## 3 Results

### 3.1 Participants

A total of 393 patients were screened, and 391 of them met the inclusion criteria and were included in the final analysis. Two patients were excluded owing to the absence of medication dispensing records. All patients were followed for survival until death or censoring on October 21, 2024.

### 3.2 Baseline characteristics

Baseline characteristics of all 391 patients are presented in **Table 1**. The median age was 64.7 years (IQR 64.1–65.9; range 39.1–81.4 years), and 58.6% were male. Most had non-squamous histology (79.0%). ECOG PS was ≥2 in 17.4%. Among all included patients, 254 patients (65.0%) received ICIs in the first-line setting, including 156 (39.9%) as mono-IT and 98 (25.1%) as chemo-IT, and 137 patients (35.0%) received ICIs in the second-line setting, including 118 (30.2%) as mono-IT and 19 (4.9%) as chemo-IT. Pembrolizumab was used in 75.7% of all patients, followed by atezolizumab (15.1%) and nivolumab (9.2%). Among those who also received chemotherapy, the most common regimen was platinum plus pemetrexed (20.7%). The median follow-up time was 40.6 months (IQR 36.6–44.6).

**Table 1:** Baseline characteristics in the total cohort of patients with mNSCLC, as well as in cohorts stratified by the timing of PPI exposure relative to ICI initiation: patients who were not prescribed PPIs from 365 days before to 30 days after ICI initiation (no PPIs −365 to +30 days of ICI initiation), patients who were prescribed PPIs exclusively in the pre-treatment period between 365 and 31 days before ICI initiation (PPIs −365 to −31 days of ICI initiation), and patients who were prescribed PPIs during the 30 days before or after ICI initiation (PPIs ±30 days of ICI initiation).

Characteristic	Total cohort N = 391 (100%)	No PPIs −365 to +30 days of ICI initiation N = 104 (100%)	PPIs −365 to −31 days of ICI initiation ^a^ N = 67 (100%)	PPIs ±30 days of ICI initiation ^b^ N = 220 (100%)
Sex, N (%)				
Male	229 58.6%	60 57.7%	47 70.1%	122 55.5%
Female	162 41.4%	44 42.3%	20 29.9%	98 44.5%
Age at ICI initiation (y), median (IQR)	64.7 (64.1–65.9)	65.3 (63.1–67.3)	65.3 (61.6–68.0)	64.7 (63.9–65.8)
Histology, N (%)				
Non-squamous	309 79.0%	78 75.0%	52 77.6%	179 81.4%
Squamous	82 21.0%	26 25.0%	15 22.4%	41 18.6%
Molecular alterations, N (%)				
No	228 58.3%	61 58.7%	40 59.7%	127 57.7%
EGFR mutations	12 3.1%	5 4.8%	1 1.5%	6 2.7%
ALK mutations	1 0.3%	/	1 1.5%	/
KRAS mutations (any)	136 34.8%	37 35.6%	21 31.3%	78 35.5%
Other ^c^	14 3.6%	1 1.0%	4 6.0%	9 4.1%
ECOG PS, N (%)				
0	53 13.6%	18 17.3%	8 11.9%	27 12.3%
1	270 69.1%	78 75.0%	51 76.1%	141 64.1%
≥2	68 17.4%	8 7.7%	8 11.9%	52 23.6%
BMI, median (IQR)	25.9 (25.3–26.6)	25.8 (24.7–27.4)	25.1 (24.0–26.1)	26.2 (25.2–27.1)
Brain metastasis at ICI initiation, N (%)				
No	332 84.9%	94 90.4%	55 82.1%	183 83.2%
Yes	59 15.1%	10 9.6%	12 17.9%	37 16.8%
Liver metastasis at ICI initiation, N (%)				
No	333 85.2%	87 83.7%	57 85.1%	189 85.9%
Yes	58 14.8%	17 16.3%	10 14.9%	31 14.1%
PD–L1 (%), median (IQR)	40 (30–60)	60 (50–75)	5 (1–60)	40 (30–60)
PD–L1 (categories), N (%)				
≥50%	186 47.6%	55 52.9%	26 38.8%	105 47.7%
1–49%	94 24.0%	18 17.3%	17 25.4%	59 26.8%
<1%	95 24.3%	23 22.1%	22 32.8%	50 22.7%
Missing	16 4.1%	8 7.7%	2 3.0%	6 2.7%
Therapy line, N (%)				
First	254 65.0%	69 66.3%	39 58.2%	146 66.4%
Second or more	137 35.0%	35 33.7%	28 41.8%	74 33.6%
Systemic cancer therapy type, N (%)				
Mono-IT	274 70.1%	77 74.0%	47 70.1%	150 68.2%
Chemo-IT	117 29.9%	27 26.0%	20 29.9%	70 31.8%
ICI drug, N (%)				
Pembrolizumab	296 75.7%	76 73.1%	47 70.1%	173 78.6%
Atezolizumab	59 15.1%	16 15.4%	8 11.9%	35 15.9%
Nivolumab	36 9.2%	12 11.5%	12 17.9%	12 5.5%
Chemotherapy, N (%)				
Pt + Pemetrexed	81 20.7%	16 15.4%	11 16.4%	54 24.5%
Pt + Paclitaxel	24 6.1%	8 7.7%	7 10.4%	9 4.1%
Pt + Paclitaxel + Bevacizumab	10 2.6%	3 2.9%	1 1.5%	6 2.7%
Pt + Gemcitabine	1 0.3%	/	/	1 0.5%
Missing	1 0.3%	/	1 1.5%	/
Follow up (months), median (IQR)	40.6 (36.6–44.6)	36.4 (33.5–39.2)	41.6 (37.5–45.7)	40.6 (36.3–44.9)
OS (months), median (IQR)	18.8 (15.2–22.4)	21.9 (14.4–29.5)	24.1 (12.1–36.1)	15.4 (10.1–20.8)

a Patients who were prescribed PPIs exclusively in the pre-treatment period had no PPI prescriptions within ±30 days of ICI initiation.

b Patients who were prescribed PPIs within ±30 days of ICI initiation could also have had PPI prescriptions between −365 and −31 days of ICI initiation.

c Other alterations include *BRAF* V600E mutations (n=7), *EGFR* exon 20 insertions (n=2), *MET* exon 14 skipping mutations (n=2), *ERBB2* exon 20 insertions (n=1), *NTRK* fusion (n=1), and *RET* mutation (n=1). BMI, body mass index; Chemo-IT, chemotherapy and immunotherapy; ECOG PS, Eastern Cooperative Oncology Group performance status; ICI, immune checkpoint inhibitor; IQR, interquartile range; mNSCLC, metastatic non-small-cell lung cancer; Mono-IT, immunotherapy monotherapy; OS, overall survival; PD-L1, programmed death–ligand 1; PPI, proton pump inhibitor; Pt, platinum-based agent (carboplatin or cisplatin); y, years.

### 3.3 PPI exposure

In the total cohort of 391 patients, PPI exposure was prevalent, with 73.4% of patients having access to PPIs between −365 and +30 days of ICI initiation (**Table 2**). Within this observation window, pantoprazole was prescribed in 230 patients (58.8%), esomeprazole in 61 (15.6%), lansoprazole in 37 (9.5%), and omeprazole in 20 (5.1%). As patients could receive more than one PPI type sequentially (rather than concomitantly), these categories are not mutually exclusive. A total of 220 patients (56.3%) had PPIs within ±30 days of ICI initiation, with the majority (47.3%,185/391) also having PPIs during the earlier −365 to −31 days of ICI initiation period. Only 67 patients (17.1%) had PPIs exclusively within −365 to −31 days prior to ICI initiation.

**Table 2:** Intensity of PPI exposure in the total cohort of patients with mNSCLC, as well as in cohorts stratified by the timing of PPI exposure relative to ICI initiation: patients who were prescribed PPIs exclusively in the pre-treatment period between 365 and 31 days before ICI initiation (PPIs −365 to −31 days of ICI initiation), and patients who were prescribed PPIs during the 30 days before or after ICI initiation (PPIs ±30 days of ICI initiation).

Characteristic of PPI exposure	Total cohort N = 391 (100%)	PPIs −365 to −31 days of ICI initiation ^a^ N = 67 (100%)	PPIs ±30 days of ICI initiation ^b^ N = 220 (100%)
Total DDDs −365 to +30 days of ICI initiation, median (IQR)	58.0 (43.0–70.0)	45.0 (30.0–70.0)	138.5 (115.5–174.0)
DDD categories −365 to +30 days of ICI initiation, N (%)			
No PPI exposure; DDD = 0	104 26.6%	/	/
Low-intensity exposure; DDD 0.1 – 159	179 45.8%	60 89.6%	119 54.1%
High-intensity exposure; DDD > 159	108 27.6%	7 10.4%	101 45.9%
Total DDDs −365 to −31 days of ICI initiation, median (IQR)	30.0 (28.0–48.0)	45.0 (30.0–70.0)	90.0 (66.5–114.5)
DDD categories −365 to −31 days of ICI initiation, N (%)			
No PPI exposure; DDD = 0	139 35.5%	/	35 15.9%
Low-intensity exposure; DDD 0.1 – 134	156 39.9%	57 85.1%	99 45.0%
High-intensity exposure; DDD > 134	96 24.6%	10 14.9%	86 39.1%
Total DDDs ±30 days of ICI initiation, median (IQR)	15.0 (5.0–24.0)	/	43.0 (37.5–50.0)
DDD categories ±30 days of ICI initiation, N (%)			
No PPI exposure; DDD = 0	171 43.7%	/	/
Low-intensity exposure; DDD 0.1-25	47 12.0%		47 21.4%
High-intensity exposure; DDD > 25	173 44.2%		173 78.6%

a Patients who were prescribed PPIs exclusively in the pre-treatment period had no PPI prescriptions within ±30 days of ICI initiation.

b Patients who were prescribed PPIs ±30 days of ICI initiation could also have had PPI prescriptions between −365 and −31 days of ICI initiation.

DDD, defined daily dose; ICI, immune checkpoint inhibitor; IQR, interquartile range; mNSCLC, metastatic non-small-cell lung cancer; PPI, proton pump inhibitor.

In the total cohort of 391 patients, the median cumulative PPI exposure across the full −365 to +30 days of ICI initiation window was 58.0 total DDDs (IQR 43.0–70.0), corresponding to approximately one DDD per week during this period. Within this timeframe, over a quarter (27.6%) of patients were exposed to more than 0.5 DDD per day and were classified as having a high-intensity exposure. Within the same observation period, the median total DDDs were highest in patients with PPIs within ±30 days of ICI initiation (138.5 total DDDs; IQR 115.5–174.0) and approximately three times lower (45.0 total DDDs; IQR 30.0–70.0) in patients with PPIs exclusively within −365 to −31 days prior to ICI initiation.

### 3.4 Overall survival

The mOS for the total cohort was 18.8 months (IQR 15.2–22.4; **Table 1**). A total of 268 deaths occurred during the follow-up period.

**Figure 1:**
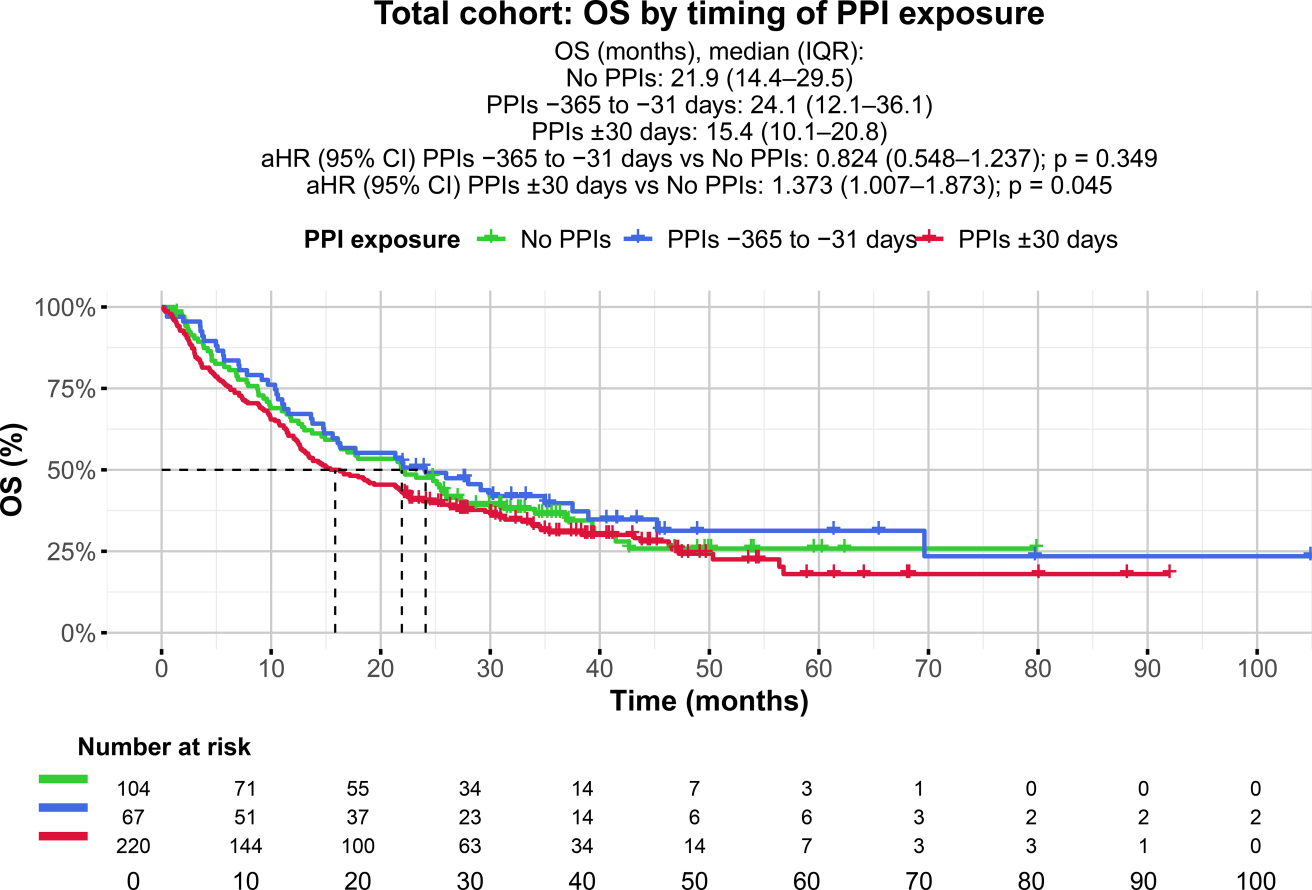
Kaplan-Meier curves showing overall survival (OS) in the total cohort (N = 391), grouped by the timing of proton pump inhibitor (PPI) exposure relative to immune checkpoint inhibitor (ICI) initiation: patients who were not prescribed PPIs from 365 days before to 30 days after ICI initiation (no PPIs −365 to +30 days of ICI initiation), patients who were prescribed PPIs exclusively in the pre-treatment period between 365 and 31 days before ICI initiation (PPIs −365 to −31 days of ICI initiation), and patients who were prescribed PPIs during the 30 days before or after ICI initiation (PPIs ±30 days of ICI initiation). Dashed lines represent the median OS for each group. Number at risk is shown below the plot.

Timing of PPI exposure was significantly associated with mOS (**Table 3**, **Figure 1**). In the multivariable Cox regression model adjusted for key prognostic factors, PPI exposure within ±30 days of ICI initiation was independently associated with worse mOS compared with no PPI exposure between −365 and +30 days of ICI initiation (mOS 15.4 vs 21.9 months, adjusted hazard ratio [aHR] 1.373; 95% CI 1.007–1.873; p = 0.045) (**Table 3**, **Figure 1**, **Supplementary Table 1**). In contrast, PPI exposure exclusively during the pre-treatment period (−365 to −31 days of ICI initiation) was not associated with mOS compared with no PPI exposure between −365 and +30 days of ICI initiation (mOS 24.1 vs 21.9 months, aHR 0.824; 95% CI 0.548–1.237; p = 0.349). Squamous histology (aHR 1.414; 95% CI 1.046–1.912; p = 0.024), lower PD-L1 expression (aHR 0.995; 95% CI 0.990–0.999; p = 0.018), second or later therapy line (aHR 1.435; 95% CI 1.018–2.023; p = 0.039), mono-IT systemic therapy (aHR 0.535; 95% CI 0.359–0.798; p = 0.002), and the presence of brain (aHR 1.631; 95% CI 1.155–2.302; p = 0.005) or liver metastases (aHR 2.626; 95% CI 1.883–3.664; p < 0.001) were also associated with worse OS (**Supplementary Table 1**).

**Table 3:** Association between the timing and intensity of PPI exposure and OS in the total cohort of patients with mNSCLC (N = 391).

PPI exposure	Total cohort				
	N = 391 (100%)				
	HR (95% CI)	p value	aHR (95% CI)	p value	OS (months), median (IQR)
PPI exposure relative to ICI initiation					
No PPIs (reference) (N = 104)	/		/	/	21.9 (14.4–29.5)
PPIs −365 to −31 days of ICI initiation ^a^ (N = 67)	0.891 (0.609–1.305)	0.555	0.824 (0.548–1.237)	0.349	24.1 (12.1–36.1)
PPIs ±30 days of ICI initiation ^b^ (N = 220)	1.149 (0.865–1.525)	0.338	1.373 (1.007–1.873)	**0.045**	15.4 (10.1–20.8)
Total DDDs −365 to +30 days of ICI initiation (N = 391)	1.000 (1.000–1.001)	0.261	1.000 (1.000–1.001)	0.338	/
DDD categories −365 to +30 days of ICI initiation					
No PPI exposure; DDD = 0; (reference) (N = 104)	/		/	/	21.9 (14.4–29.5)
Low-intensity exposure; DDD 0.1 – 159; (N = 179)	0.956 (0.710–1.287)	0.766	1.075 (0.782–1.477)	0.656	22.1 (14.0–30.2)
High-intensity exposure; DDD > 159; (N = 108)	1.336 (0.969–1.843)	0.077	1.454 (1.023–2.067)	**0.037**	13.4 (10.4–16.4)
Total DDDs −365 to −31 days of ICI initiation (N = 391)	1.001 (1.000–1.001)	0.293	1.000 (0.999–1.001)	0.385	/
DDD categories −365 to −31 days of ICI initiation					
No PPI exposure; DDD = 0; (reference) (N = 139)	/		/	/	21.9 (15.1–28.7)
Low-intensity exposure; DDD 0.1 – 134; (N = 156)	0.981 (0.740–1.300)	0.894	0.983 (0.730–1.325)	0.913	21.4 (12.8–30.0)
High-intensity exposure; DDD > 134; (N = 96)	1.366 (1.004–1.858)	**0.047**	1.331 (0.956–1.853)	0.091	13.6 (10.2–17.0)
Total DDDs ±30 days of ICI initiation (N = 391)	1.002 (0.998–1.005)	0.351	1.002 (0.998–1.005)	0.369	/
DDD categories ±30 days of ICI initiation					
No PPI exposure; DDD = 0; (reference) (N = 171)	/		/	**/**	22.1 (16.5–27.7)
Low-intensity exposure; DDD 0.1–25; (N = 47)	1.361 (0.938–1.975)	0.104	2.234 (1.503–3.321)	**<0.001**	13.8 (7.7–19.9)
High-intensity exposure; DDD > 25; (N = 173)	1.160 (0.896–1.502)	0.259	1.343 (1.013–1.780)	**0.04**	16.6 (10.7–22.5)

a Patients who were prescribed PPIs exclusively in the pre-treatment period between 365 and 31 days before ICI initiation (−365 to −31 days of ICI initiation) had no PPI prescriptions within ±30 days of ICI initiation.

b Patients who were prescribed PPIs during the 30 days before or after ICI initiation (± 30 days of ICI initiation) could also have had PPI prescriptions between −365 and −31 days of ICI initiation.

Univariable Cox regression is reported with HR (95% CI) and multivariable with aHR (95% CI), assessing each PPI exposure as the dependent variable and adjusting for sex, age, BMI, ECOG PS, histology (non-squamous/squamous), brain and liver metastases, PD-L1 (%), therapy line, and systemic cancer therapy type. Bold *p* values indicate statistically significant differences.

(a)HR, (adjusted) hazard ratio; DDD, defined daily dose; ECOG PS, Eastern Cooperative Oncology Group performance status; ICI, immune checkpoint inhibitor; mNSCLC, metastatic non-small-cell lung cancer; OS, overall survival; PPI, proton pump inhibitor.

**Figure 2:**
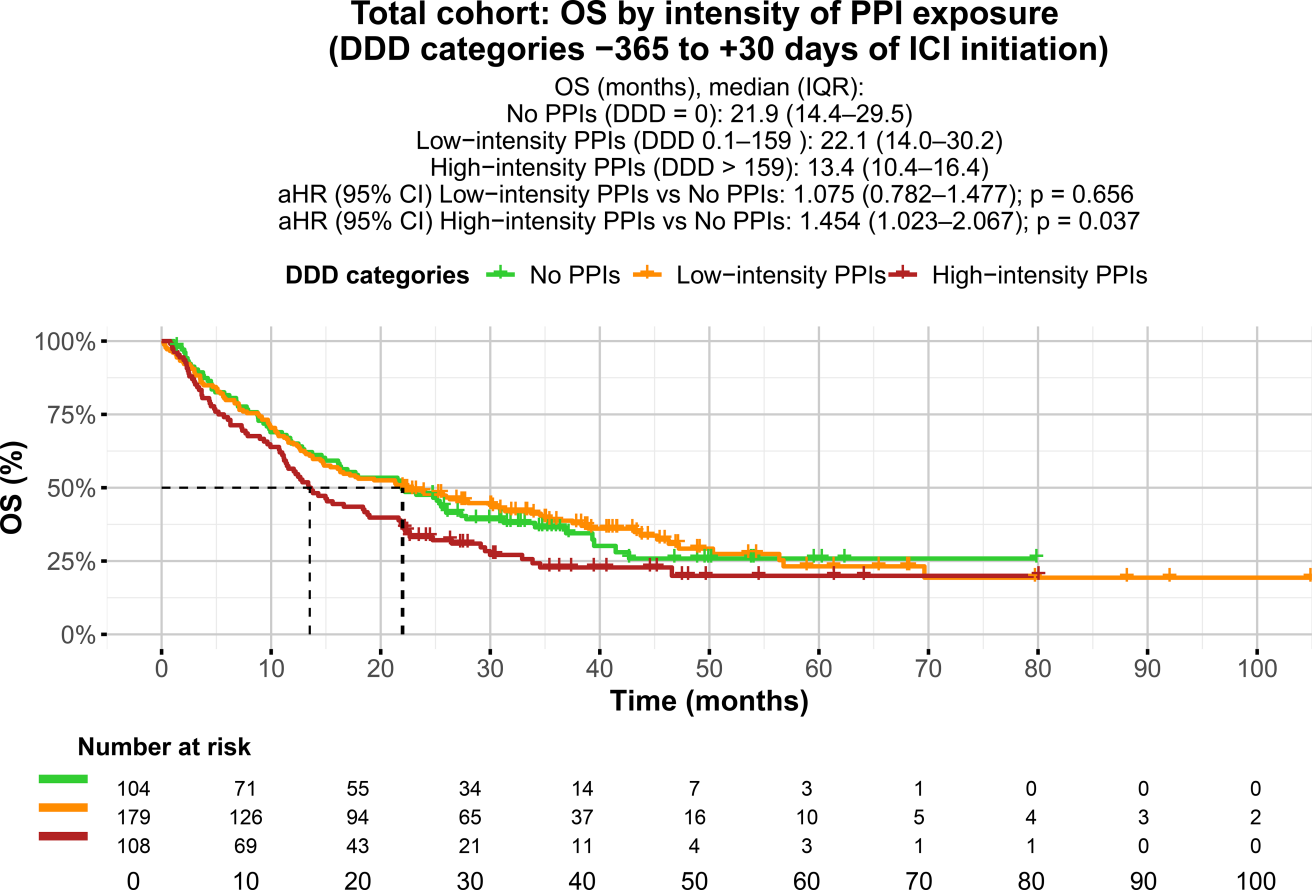
Kaplan-Meier curves showing overall survival (OS) in the total cohort (N = 391), grouped by the intensity of proton pump inhibitor (PPI) exposure, measured in total defined daily doses (DDDs), during the observation window from 365 days before to 30 days after immune checkpoint inhibitor (ICI) initiation: patients with no PPI exposure (DDD = 0), patients with low-intensity exposure (DDD 0.1–159), and patients with high-intensity exposure (DDD >159). Dashed lines represent the median OS for each group. Number at risk is shown below the plot.

Both high and low intensities of PPI exposure within ±30 days of ICI initiation were significantly associated with OS, compared with no PPI exposure during the same time frame (mOS 16.6 vs 22.1 months, aHR 1.343; 95% CI 1.013–1.780; p = 0.040, and mOS 13.8 vs 22.1 months, aHR 2.234; 95% CI 1.503–3.321; p < 0.001; **Table 3**). The intensity of PPI exposure between −365 and +30 days of ICI initiation was also significantly associated with mOS (**Table 3**, **Figure 2**). Patients with high-intensity exposure (DDD > 159) had worse mOS compared with those with no PPI exposure between −365 and +30 days of ICI initiation (mOS 13.4 vs 21.9 months, aHR 1.454; 95% CI 1.023–2.067; p = 0.037) (**Table 3**, **Figure 2**, **Supplementary Table 2**). Low-intensity exposure (DDD 0.1–159) was not associated with mOS compared with no PPI exposure between −365 and +30 days of ICI initiation (mOS 22.1 vs 21.9 months, aHR 1.075; 95% CI 0.782–1.477; p = 0.656). However, when total PPI exposure was analyzed as a continuous variable, no significant association with mOS was observed in any of the predefined time windows (Cox proportional hazards models p > 0.05; **Table 3**).

## 4 Discussion

In this retrospective cohort study of 391 mNSCLC patients treated with ICIs, significant associations were observed between mOS and both the timing and intensity of PPI exposure. Patients with PPI exposure within 30 days before or after the start of ICI therapy had significantly worse mOS compared with those without any PPI exposure from 365 days before to 30 days after treatment initiation. Moreover, our findings suggest for the first time in mNSCLC that high-intensity PPI exposure during the full observation window, defined as an average intake exceeding 0.5 DDDs, was independently associated with reduced mOS compared to no PPI exposure.

### 4.1 PPI exposure

PPI exposure was highly prevalent in our cohort. Close to ICI initiation, within 30 days before or after the start of ICI therapy, 56.3% of patients had access to PPI, and the majority had also been exposed one year prior, indicating that PPI exposure near the start of ICI often reflected prior long-term use. This prevalence is substantially higher than previously reported in *post hoc* analyses of randomized clinical trials, in which approximately one-third of mNSCLC patients were exposed to PPIs in the same observational window (29, 30). In contrast, several real-world retrospective studies have reported much higher rates, with up to 84% of cancer patients being PPI users, with the highest prevalence observed in thoracic cancer populations (31–33). These findings are expected, as patients treated within routine clinical practice are more complex and prescribing practices are more variable than those in the more rigorous clinical trial setting (39).

Our study adds to earlier evidence on the characteristics of PPI use in patients treated with ICI by extending the exposure window from only 30–60 days to one year prior to ICI start, providing a more comprehensive assessment of long-term PPI exposure. In our cohort, PPI exposure was very common across the full observation window, with 73.4% of patients having access to PPI from one year before to 30 days after ICI initiation. Evidence on long-term PPI exposure in this context is limited to a single real-world study reporting a 45% prevalence of PPI exposure within one year prior to immunotherapy in lung cancer and melanoma patients, with the lower prevalence possibly reflecting a more proactive approach to deprescribing of unnecessary medicines in that setting (40, 41).

In our study, PPI treatment intensity was quantified by DDD, a standardized measure of cumulative exposure that facilitates comparisons between studies. High-intensity PPI exposure one year prior to ICI start (DDD > 159) occurred in 27.6% of patients, corresponding to a therapeutic dose (e.g., pantoprazole 40 mg/d) for at least half, or a gastroprotective dose (e.g., pantoprazole 20 mg/d) for the full duration of the observation period. As most PPI indications require only 4–8 weeks of therapy (28–56 DDDs) (41), this finding suggests frequent chronic or potentially excessive use of PPIs. Overall, our findings indicate that, in patients with mNSCLC, PPI use is not only more prevalent but frequently chronic and intensive over the year preceding ICI start.

### 4.2 Association of PPI exposure with OS

In our cohort, patients with PPI exposure within 30 days before or after the start of ICI therapy had a 37% increased risk of death compared with those without any PPI exposure from one year before to 30 days after treatment initiation (aHR 1.373; 95% CI 1.007–1.873; p = 0.045). The period around ICI initiation seems to be vital as both low- and high-intensity PPI exposure within 30 days before or after the start of ICI therapy were associated with worse mOS compared with no PPI exposure during the same period (aHR 2.234; 95% CI 1.503–3.321; p < 0.001, and aHR 1.343; 95% CI 1.013–1.780; p = 0.040, respectively). Previous studies (29–31) have consistently reported that PPI exposure in this observation window is associated with poorer mOS in mNSCLC patients, and similar associations have been reported for slightly broader windows near the start of ICI (32). Evidence on a dose–response effect during this period remains mixed; while some authors report worse mOS only with therapeutic PPI doses (42), others have found no dose–response relationship (43). Our findings reinforce existing evidence, emphasizing the ±30-day window around ICI initiation as a critical period during which PPI use should be completely avoided whenever clinically feasible. A growing body of translational research provides mechanistic support for this observation. Gastric acid suppression facilitates oral-to-gut microbial transmission, promoting the expansion of oral species in the lower gastrointestinal tract (25, 26, 44). PPIs also directly inhibit gut bacteria and reduce overall microbial diversity, including depletion of commensal species linked to favorable ICI responses (26, 27). Although definitive prospective randomized studies evaluating the impact of concomitant medicines on the gut microbiota in immuno-oncology may never be available, expert consensus underscores the importance of minimizing non-essential medicines and avoiding unnecessary polypharmacy in patients receiving ICIs (25).

Only 67 patients were exposed to PPIs within one year prior but not within 30 days before or after the start of ICI therapy. Exposure to PPIs within this observation window was not associated with mOS compared with no PPI exposure (aHR 0.824; 95% CI 0.548–1.237; p = 0.349). However, most of these patients had low treatment intensity (median total DDDs 45.0; IQR 30.0–70.0), and only 10 met the criteria for high-intensity exposure. This lack of association between PPI exposure within one year prior, but not within 30 days before or after the start of ICI therapy, and mOS, may be due to the small sample size and lower cumulative exposure in that subgroup, rather than a true absence of risk. Supporting this interpretation, Eng et al. (45) reported that PPI use during the year prior to ICI initiation was associated with worse mOS (aHR 1.21; 95% CI 1.03-1.42; p = 0.02), with even greater risk when PPI use was combined with antibiotic use (aHR 1.33; 95% CI 1.16–1.52; p < 0.001). Notably, antibiotic use alone in the year preceding ICI therapy was also linked to poorer survival outcomes (28, 45). Future research should investigate whether the discontinuation of long-term PPI use 30 days prior to ICI initiation may eliminate the detrimental effect of PPIs on ICI outcomes.

We provide valuable new evidence that cumulative PPI exposure during the year preceding ICI therapy may hamper ICI effectiveness, with higher long-term exposure posing increased risk. High-intensity PPI exposure (DDD > 159) from one year before to 30 days after treatment initiation was independently associated with a 45% increased risk of death compared with no PPI exposure in this period (aHR 1.454; 95% CI 1.023–2.067; p = 0.037), whereas low-intensity exposure was not associated with mOS. Although direct evidence for intensity over a one-year timeframe in mNSCLC is still emerging, Eng et al. (40) reported similar findings in one study that showed a dose-dependent effect based on weeks of PPI exposure in the year prior to ICI initiation in cancer patients (aHR 1.00 per week; 95% CI 1.00-1.01; p = 0.05). Given the widespread use of PPIs, their potential to alter the gut microbiota may be more pronounced than that of other medicines; therefore, these results highlight the need for greater caution in PPI prescribing practices.

### 4.3 Implication for clinical practice

While current research focuses on restoring gut microbial diversity through interventions such as probiotics or fecal microbiota transplantation (46, 47), a preventive approach could instead target dysbiosis at its source by optimizing the use of medicines known to affect the gut microbiota, including deprescribing PPIs. Since up to 65% of PPI prescriptions may be inappropriate (48, 49) and because PPI exposure near ICI initiation is associated with worse mOS (29–31), early PPI deprescribing, ideally at least one month before systemic therapy, is urgent (41, 50–52). Deprescribing at the time of ICI initiation may be too late, and efforts should therefore focus on discontinuing PPIs earlier, possibly at the time of cancer diagnostics, when patients are first evaluated for suspected malignancy. Current evidence indicates that PPIs should be deprescribed at least 30 days before and withheld for at least 30 days after starting immunotherapy. Furthermore, as both abrupt discontinuation and gradual tapering of PPIs are equally supported by deprescribing protocols (41, 50–52), immediate cessation may be preferable in oncology patients approaching systemic treatment with ICIs. In such cases, particularly in patients with mild or nonspecific gastrointestinal symptoms, alternative approaches should be considered, including non-pharmacologic measures such as dietary modifications, avoiding late meals, and head-of-bed elevation and short-term use of antacids or H2-receptor antagonists which are expected to have a less pronounced impact on the gut microbiota (41, 44).

While deprescribing PPIs close to ICI initiation is a critical target, our findings also suggest that high cumulative PPI exposure in the year preceding ICI initiation is associated with worse OS. Considering that future immunotherapy eligibility is rarely predictable a year in advance, these findings support rational PPI prescribing at the population level to help reduce downstream risks for patients who may ultimately receive ICIs.

### 4.4 Limitations

This study has several limitations inherent to its retrospective design. Baseline imbalances between exposure groups are expected in such studies and limit comparability, introducing potential bias despite multivariable adjustment. Prescription records do not confirm actual patient use of medicine; in addition, over-the-counter PPI use, although expected to be minimal, could not be accounted for. Residual confounding from unmeasured variables, such as concurrent use of antibiotics or corticosteroids, remains possible. The analysis was based on data from a single academic oncology center, with a limited number of patients in some observation windows, potentially limiting the generalizability of the findings to broader healthcare settings. Gut microbiota composition was not directly assessed, which limits interpretation of the observed associations. Consequently, the findings indicate association rather than causation and should be further explored, ideally through prospective randomized deprescribing trials.

## 5 Conclusion

Both the timing and intensity of PPI exposure are important and potentially modifiable factors associated with reduced mOS in mNSCLC patients receiving ICIs. PPI exposure within one month before or after ICI initiation, as well as high-intensity exposure in the year preceding treatment, were significantly associated with poorer survival outcomes. These findings highlight the need for a stringent evaluation of the benefits and harms of PPI therapy, not only in each oncology patient but also at the population level, to ensure that breakthrough therapies such as cancer immunotherapy can extend survival to their fullest potential.

## Data Availability

The original contributions presented in the study are included in the article/**Supplementary Material**. Further inquiries can be directed to the corresponding author/s.
