# Supplementary material for: Timing and intensity of proton pump inhibitor exposure hampers overall survival in patients with metastatic non-small cell lung cancer treated with immune checkpoint inhibitors: a retrospective cohort study
Source: Front Immunol. 2026 Jan 28;17:1682723. doi: 10.3389/fimmu.2026.1682723 (PMC12891114; doi:10.3389/fimmu.2026.1682723)
Supplement: Supplementary file 1 [file DataSheet1.docx]

Supplementary Material

# Supplementary Tables

**Supplementary Table 1** Multivariable analysis of the association between the timing of PPI exposure relative to ICI initiation and OS in the total cohort of patients with mNSCLC (N = 391; 17 missing cases).

| **Characteristics** | **B** | **SE** | **aHR**  **(95% CI)** | **p value** |
| --- | --- | --- | --- | --- |
|  |  |  |  |  |
| **Sex**  Male (reference)  Female | 0.124 | 0.133 | 1.132 (0.872–1.470) | 0.353 |
| **Age at ICI initiation** (y) | 0.014 | 0.009 | 1.014 (0.996–1.033) | 0.134 |
| **Histology**  Non-squamous (reference)  Squamous | 0.346 | 0.154 | 1.414 (1.046–1.912) | **0.024** |
| **PD–L1** (%) | -0.005 | 0.002 | 0.995 (0.990–0.999) | **0.018** |
| **Therapy line**  First (reference)  Second or more | 0.361 | 0.175 | 1.435 (1.018–2.023) | **0.039** |
| **Systemic cancer therapy type**  Mono-IT (reference)  Chemo-IT | -0.625 | 0.204 | 0.535 (0.359–0.798) | **0.002** |
| **ECOG PS**  0 (reference) vs 1 | 0.240 | 0.207 | 1.271 (0.847–1.906) | 0.247 |
| **ECOG PS**  0 (reference) vs ≥2 | 0.393 | 0.241 | 1.481 (0.923–2.377) | 0.103 |
| **BMI** | -0.014 | 0.013 | 0.986 (0.961–1.011) | 0.272 |
| **Brain metastasis at ICI initiation**  No (reference)  Yes | 0.489 | 0.176 | 1.631 (1.155–2.302) | **0.005** |
| **Liver metastasis at ICI initiation**  No (reference)  Yes | 0.966 | 0.170 | 2.626 (1.883–3.664) | **<0.001** |
| **PPI exposure**  No PPIs −365 to +30 days of ICI initiation (reference) vs PPIs −365 to −31 days of ICI initiation ^a^ | -0.194 | 0.207 | 0.824 (0.548–1.237) | 0.349 |
| **PPI exposure**  No PPIs −365 to +30 days of ICI initiation (reference) vs PPIs ±30 days of ICI initiation ^b^ | 0.317 | 0.158 | 1.373 (1.007–1.873) | **0.045** |

^a^ Patients who were prescribed PPIs exclusively in the pre-treatment period between 365 and 31 days before ICI initiation (−365 to −31 days of ICI initiation) had no PPI prescriptions within ±30 days of ICI initiation.

^b^ Patients who were prescribed PPIs during the 30 days before or after ICI initiation (±30 days of ICI initiation) could also have had PPI prescriptions between −365 and −31 days of ICI initiation.

Bold p values indicate statistically significant differences.

(a)HR: (adjusted) hazard ratio; B: regression coefficient; BMI: body mass index; Chemo-IT: chemotherapy and immunotherapy; ECOG PS: Eastern Cooperative Oncology Group performance status; ICI: immune checkpoint inhibitor; IQR: interquartile range; mNSCLC: metastatic non-small-cell lung cancer; Mono-IT: immunotherapy monotherapy; OS: overall survival; PD-L1: programmed death–ligand 1; PPI: proton pump inhibitor; SE: standard error; y: years.

**Supplementary Table 2** Multivariable analysis of the association between the intensity of PPI exposure from 365 days before to 30 days after ICI initiation (−365 to +30 days of ICI initiation) and OS in the total cohort of patients with mNSCLC (N = 391; 17 missing cases).

| **Characteristics** | **B** | **SE** | **aHR**  **(95% CI)** | **p value** |
| --- | --- | --- | --- | --- |
| **Sex**  Male (reference)  Female | 0.176 | 0.132 | 1.192 (0.920–1.545) | 0.183 |
| **Age at ICI initiation** (y) | 0.013 | 0.009 | 1.013 (0.994–1.032) | 0.175 |
| **Histology**  Non-squamous (reference)  Squamous | 0.338 | 0.154 | 1.402 (1.037–1.895) | **0.028** |
| **PD–L1** (%) | -0.005 | 0.002 | 0.995 (0.990–0.0999) | **0.027** |
| **Therapy line**  First (reference)  Second or more | 0.317 | 0.175 | 1.373 (0.973–1.935) | 0.071 |
| **Systemic cancer therapy type**  Mono-IT (reference)  Chemo-IT | -0.615 | 0.202 | 0.540 (0.364–0.804) | **0.002** |
| **ECOG PS**  0 (reference) vs 1 | 0.199 | 0.207 | 1.221 (0.813–1.832) | 0.336 |
| **ECOG PS**  0 (reference) vs ≥2 | 0.401 | 0.242 | 1.493 (0.930–2.397) | 0.097 |
| **BMI** | -0.013 | 0.013 | 0.987 (0.962–1.012) | 0.304 |
| **Brain metastasis at ICI initiation**  No (reference)  Yes | 0.403 | 0.175 | 1.496 (1.062–2.108) | **0.021** |
| **Liver metastasis at ICI initiation**  No (reference)  Yes | 0.907 | 0.168 | 2.477 (1.781–3.444) | **<0.001** |
| **DDD categories −365 to +30 days of ICI initiation**  DDD = 0 (reference) vs  DDD 0.1 – 159 | 0.072 | 0.162 | 1.075 (0.782–1.477) | 0.656 |
| **DDD categories −365 to +30 days of ICI initiation**  DDD = 0 (reference) vs  DDD > 159 | 0.374 | 0.180 | 1.454 (1.023–2.067) | **0.037** |

Bold p values indicate statistically significant differences.

(a)HR: (adjusted) hazard ratio; B: regression coefficient; BMI: body mass index; Chemo-IT: chemotherapy and immunotherapy; DDD: defined daily dose of PPIs; ECOG PS: Eastern Cooperative Oncology Group performance status; ICI: immune checkpoint inhibitor; IQR: interquartile range; mNSCLC: metastatic non-small-cell lung cancer; Mono-IT: immunotherapy monotherapy; OS: overall survival; PD-L1: programmed death–ligand 1; PPI: proton pump inhibitor; SE: standard error; y: years.
